# “Children get sick all the time”: A qualitative study of socio-cultural and health system factors contributing to recurrent child illnesses in rural Burkina Faso

**DOI:** 10.1186/s12889-016-3067-0

**Published:** 2016-05-10

**Authors:** Lise Rosendal Østergaard, Pia Juul Bjertrup, Helle Samuelsen

**Affiliations:** Department of Anthropology, University of Copenhagen, Øster Farimagsgade 5, DK-1353 Copenhagen K, Denmark

**Keywords:** Treatment seeking strategies, Children, Quality of care, Health care system, Burkina Faso

## Abstract

**Background:**

In Burkina Faso, the government has implemented various health sector reforms in order to overcome financial and geographical barriers to citizens’ access to primary healthcare throughout the country. Despite these efforts, morbidity and mortality rates among children remain high and the utilization of public healthcare services low. This study explores the relationship between mothers’ intentions to use public health services in cases of child sickness, their social strategies and cultural practices to act on these intentions and the actual services provided at the primary health care facilities. Focusing on mothers as the primary caregivers, we follow their pathways from the onset of symptoms through their various attempts of providing treatment for their sick children. The overall objective is to discuss the interconnectedness of various factors, inside and outside of the primary health care services that contribute to the continuing high child morbidity and mortality rates.

**Methods:**

The study is based on ethnographic fieldwork, including in-depth interviews and follow-up interviews with 27 mothers, informal observations of daily-life activities and structured observations of clinical encounters. Data analysis took the form of thematic analysis.

**Results and discussion:**

Focusing on the mothers’ social strategies and cultural practices, three forms of responses/actions have been identified: home-treatment, consultation with a traditional specialist, and consultation at the primary health care services. Due to their *accumulated vulnerabilities*, mothers shift pragmatically from one treatment to another. However, the sporadic nature of their treatment-seeking hinders them in obtaining long-term solutions and the result is recurrent child illnesses and relapses over long periods of time. The *routinization* of the clinical encounter at rural dispensaries furthermore fails to address these complexities of children’s illnesses.

**Conclusions:**

The analysis of case studies, interviews and observations shows how mothers in a rural area struggle and often fail to receive care at public healthcare facilities. Health service delivery could be organized in a manner that responds better to the needs of these mothers in terms of both access and retention.

## Background

“Children fall sick all the time” was an expression we often heard from mothers when talking about everyday village life in Burkina Faso. The burden of diseases among children in this region is high and poverty driven. Poverty is especially widespread in rural areas and is characterized by limited access to basic social services. In Burkina Faso, the estimated under-five mortality rate is 98 [1] with malaria, bacterial and respiratory infections as the leading causes of child death. While a number of other low-income societies have managed to reduce the child mortality rates considerably, Burkina Faso remains in the group of countries with highest under-five mortality rates [2]. Malaria is endemic and perennial and peaks in the rainy season, from July to September. In recognition of the high burden of malaria morbidity and mortality, artemisinin combination therapies are subsidized and the management of severe malaria has been free of charge since 2005. In addition, insecticide-treated bed nets have also been distributed free of charge since 2010 [3]. In most rural health dispensaries, the rapid diagnostic test for malaria is available. Availability and access have been documented as important factors for utilization of public health care facilities [4, 5]. The government of Burkina Faso has, during the last 15 to 20 years, focused on improvements in geographical and financial access to primary health care across the country [5–7]. In other words, the healthcare system in Burkina Faso has to a certain extent addressed issues of availability and accessibility. However, no effective mechanism to protect the poorest from the burden of catastrophic expenditure is operational [5]. By contrast to other countries in the sub-region, Burkina Faso has opted for partial over full subsidy of maternal health services thus requiring a co-payment of 20 % by families for targeted services [8]. One contributing factor to low utilization of health services by the poorest population groups is “out-of-pocket expenditure”, in terms of payments for medicine, consultations and hospitalization as well as indirect costs such as lost working time and transportation [7, 9]. Another contribution factor is the often strained relationship between nurses and midwives and their patients in public healthcare facilities in Burkina Faso [10–12]. Combined these factors contribute to patient delay and poor retention as shown in a number of studies on quality of care in public health facilities in Sub-Saharan Africa [13–16].

It is well established by anthropologists and health service researchers that many of the efforts to improve the quality of public healthcare have taken the perspective of those who provide healthcare rather than of those who use, or choose not to use, public healthcare [17–19]. Approaches to low utilization and delay are often framed within the epistemology of biomedicine and have been criticized for failing to include ordinary people’s experiences of sickness and their accounts of whether and when they decide to seek help from state-provided health care services [20–24].

In this article we wish to respond to the call made by Biehl and Petryna [24] for more “peopled accounts” of the way public health care is being utilized with detailed, context-specific information from rural Burkina Faso. Being the primary caregivers in the household, women are the most frequent users of public health care, from which it follows that the women’s perspectives are critical to furthering our understanding of what could be done to improve the quality of public healthcare. This study is a part of a collective research project involving the universities of Ouagadougou and Copenhagen in exploring how people in rural areas make use of and experience government health services. Taking the perspectives of mothers, we track their health seeking practices in the context of child sickness. We analyze their acts of interpreting and responding to bodily signs of sickness in children and the implications for utilization of available state-provided health care. In contrast to other studies of treatment seeking and patient delays, we have not followed a specific disease. As the objective of our study is to analyze how women living in difficult situations make use of public healthcare in general, we have chosen to track their actions and inactions, from the moment they discover their child’s first symptoms, regardless of which diagnosis is attributed later on.

## Methods

### Study area

The data collection took place in a village of approximately 2,800 inhabitants located 25 km away from the regional capital Tenkodogo. The major ethnic groups are Bissa, Mossi and Fulani. Most people in this village are subsistence farmers. In this region the estimate is that 75,6 % of women have not been to school, compared to a national average of 69,8 % [25]. People’s access to biomedical healthcare consists of two state-sponsored healthcare centers (located six kilometers from each other) and the regional hospital 30 km away. The nearest private facility is a small clinic in Tenkodogo but it was not used by village people due to high costs of services. Pharmaceuticals, however, can be bought at the local market where medicine hawkers sell paracetamol, antibiotics and antimalarial medicines etc. by the tablet. In addition, a number of folk practitioners/traditional healers such as bonesetters, herbal specialists and marabouts (Islamic healers) offer health services in relation to specific symptoms or diseases.

### Data collection

Ethnographic data were collected by all three authors in 2012 and 2013, with follow-up visits in 2014. The data comes from a combination of many conversations with mothers, informal observations of daily-life activities and structured observations of clinical encounters at the healthcare center. A group of 27 women were followed over a minimum period of five months. Through repeated visits to their homes we and our interpreters managed to build up a certain trust base that allowed us to gain insight into their broader concerns rather than just those related to health. Our presence in the field was critical to building up familiarity with our interlocutors: HS has worked and conducted fieldwork recurrently over a period of 16 years in the area; PJB lived in the village for five months; and LRØ lived in the regional town, Tenkodogo, approximately 25 km from the village, for seven months, with shorter follow-up visits. Our interlocutors were recruited both from a survey conducted as a part of HS’s earlier fieldwork and through our participation in the field using snowballing techniques. Although the interviewees are not representative of Burkinabe mothers the group includes variations in religion (Muslims, Catholics), ethnicity (Bissa, Mossi, Fulani) and level of education (none, primary school). Some but not all of our interlocutors spoke French. All lived in a situation of restrained material and financial resources, as most rural subsistence farmers in Burkina Faso do. Interviews were conducted with the assistance of two female and one male interpreter.

We asked the women in our sample about childhood diseases, therapeutic preferences and experiences of interactions with the staff at the local healthcare center. The interviews were complemented by six focus groups covering women’s experiences with the different forms of healthcare services. LRØ conducted structured observations of maternal and child consultations in two healthcare centers close to the village, complemented with in-depth interviews with the nurses and midwives working there.

Observations were recorded in detailed field notes. Most but not all interviews and focus groups discussions were audio-recorded, translated and transcribed. Those that were not in French were translated by a team of professional translators and quality checked by an independent Burkinabe health researcher.

### Ethics

Ethical permission for this study was granted by the Ethical Committee of the Ministry of Health and by the Burkina Faso Ministry of Scientific Research and Innovation and the Regional Directorate of Health [Reference number 2013-00000181/MRSI/SG/CNRST/DG/DS). Furthermore, with our interlocutors we explained the study purposes as well as the fact that it was possible and acceptable to withdraw from the interview, before obtaining their verbal consent [26]. During participant observation several episodes of child illness occurred that required ethical reflection and practical engagement and if mothers asked for our advice we did not remain neutral observers. When the illness seemed severe we encouraged them to go to the healthcare center or the referral hospital. In several cases we offered assistance in terms of transport. In a few situations we paid the bill and asked questions on behalf of the mothers, for instance, when a mother was being informed by hospital staff that no supplies of blood were available for her child. We chose to intervene based on a case-by-case judgment about what seemed to be the right thing to do.

### Data analysis

We adopted a reflexive approach to data analysis and used thematic analysis in that we have looked for themes across our data which could provide insights into the mothers’ vulnerabilities [27]. Themes stood out inductively from interviews and were compared with notes from observations. Quotes from the interviews and focus group discussions have been used where they constitute accounts of vulnerabilities shared by the interviewed women. We foreground the case of one woman, Adrienne (the name is a pseudonym), in order to show how she responded to the problems of child health. This emphasis on empirical particularity offers insights into the circumstances of health seeking practices and is compared to data from interviews, focus groups and observations.

Inspired by Ribera and Hausmann-Muela [28] we employ the notion of *accumulated vulnerabilities* in the analysis of the mother’s social and economic situation. It captures processes whereby penury, hardship and problems accumulate and reinforce each other. Vulnerability is linked to demand for health services in that illness, social and economic vulnerability and care-seeking practices affect each other. We furthermore analyze and discuss the *routinization* of the clinical encounter with its basic logic of “reading” the symptoms – prescribing treatment – expecting healing. Routinization of healthcare is a matter of delivering standardized services at the expected level of quality [29]. The health workers “process” patients using standardized procedures of diagnosing them, treating them and enabling them to return to productive life. We finally discuss how the rural healthcare facilities – as well as the mothers – are challenged by the accumulated vulnerabilities and the incapacity of the health services to address the complex and often chronic conditions of co-morbidity small children in the study area suffer from.

## Results

### The mothers’ perspectives

Almost all the interviewed women expressed a preference for state-organized healthcare over other forms of treatment when their children were sick. Following 27 mothers and observing cases of childhood illnesses in their families discloses different domains of vulnerability influencing the mothers’ therapeutic trajectories. All the interlocutors expressed some kind of vulnerability in terms of resources, worrying about the cost of therapy, both direct (financial cost) and indirect cost (lost working time). Furthermore, the mothers’ felt that they were socially vulnerable during the clinical encounter as they were anxious about how they would get attention from the health professionals.

### A case study: the daughter of Adrienne

Over a three-month period, Adrienne’s six-year-old twins and her two-year-old daughter suffered from consecutive episodes of fever, diarrhea, malnutrition and bronchitis. Adrienne is 34 years old and lives in a small compound with her husband, their six children, her mother-in-law and a widowed sister-in-law with her three children. The family has a small plot of land for farming to supplement her husband’s unstable earnings. Adrienne tends a *cabaret* (bar) in the courtyard where millet beer is prepared, sold and consumed. The following description offers insights into care-seeking for Adrienne’s daughter who suffered from repeated and unsolved episodes of illness (see Table 1 for a detailed timeline of her illness episodes). At one of our visits in Adrienne’s household in May, 2013, it is clear that Adrienne’s daughter has still not recovered. The little girl’s condition keeps changing from moments of improvement to relapses into sickness again. Adrienne has been giving her herbal medicines at home as well as medicines prescribed at the dispensary in the neighboring village and at the regional hospital. Sometimes the girl is able to leave the compound and play with the other children, even though her small body still suffers from fever, fatigue, and malnutrition. At other times she is plagued with fever, cramps and vomiting and Adrienne places her on a plastic mat in the shade of a tree in the compound. One Friday morning, after Adrienne has tended to her sick child, who was vomiting and shivering all night, PJB suggested taking them to the hospital, assuming that transport and costs were her main concerns. We were surprised to find out that Adrianne was reluctant to go as the following day was the day of her *cabaret*.

**Table 1:** Timeline covering a 6-year-old girl’s sickness episodes over three months

Day 1	Consultation at the local healthcare center, diagnosed with malaria and prescribed artemisinin
Day 4	Consultation at healthcare center in the neighboring village, 6 km away, diagnosed with anemia and referred to the Regional Hospital
Day 5	Hospitalized with severe malaria in the Regional Hospital, 30 km away, self-organized transportation, treated for malaria, anemia and bacterial infection
Day 6	Hospitalized at the Regional Hospital (with mother and siblings)
Day 17	Sleepless night with high fever
Day 20	Hospitalized at the healthcare center (with mother and baby sister)
Day 21	Hospitalized at the healthcare center (with mother and baby sister)
Day 23	Referred to the Regional Hospital, diagnosed with simple malaria
Day 31	Sleepless night with high fever. Home treatment with traditional medicines bought at the market
Day 32	Self-referral to Regional hospital, diagnosed with severe bronchitis and malaria
Day 33	Hospitalized
Day 34	Hospitalized
Day 35	Hospitalized
Day 35	Consultation with traditional healer who provided protective scarifications on chest and administered herbal medicine.
Day 41	Follow-up consultation at Regional Hospital. Sent home without consultation since the doctor is not available
Day 71	Consultation at the healthcare center in the neighboring village for malnourished children with siblings. Received portion of fortified food for 2 weeks
Day 73	Follow-up visit at healthcare center in neighboring village
Day 84	Consultation at the healthcare center in the neighboring village for malnourished children with siblings. Nurses leave before she is treated and mother is told to come back the following day
Day 86	Fever. Home-treatment with paracetamol. Consultation at the healthcare center in the neighboring village for malnourished children with siblings. Received portion of fortified food for 2 weeks
Day 87	Home-treatment with paracetamol

Summary of the mother’s account and as reported in the child’s health booklet

Adrienne’s vulnerability was also related to her husband’s alcohol problems; his low social status fell back upon Adrienne and despite the widespread sociality that she participated in, she could not count on support from neighbors nor from her most intimate social relations. Her sister-in-law, who suffered in secrecy from a chronic disease, was living in poverty herself. She complained about how Adrienne failed to share meals among the two households, thereby destabilizing the reciprocity involved in the sharing of food in many African countries [30]. We observed how she struggled to access care, some of the time with support, most of the time without. Because of time spent on healthcare she had often lost income from the *cabaret*. To cope with that, Adrienne had to use her small savings and accept help from the foreign researchers. Adrienne’s case offers a gist of the vulnerability in which many of the women live.

### Living with poverty – strategies to mobilize scarce resources

A key factor in understanding the context of vulnerability which conditions care-seeking is women’s limited access to cash. Although healthcare for children under five is subsidized by the state, even the small contribution that patients have to make towards the cost for hospitalizations, tests and medicines is a matter of concern for families, and the mothers know that they have to bring money to the consultation. During the focus group discussions, women from more robust households than Adrienne’s, especially those with their own income, reported that they spent most or all of their money on food, health and clothing with limited opportunities to save any of it. Our interlocutors have few other means of earning money themselves because of unequal access to land (women had smaller plots of land compared to men) and labor (women could only rely on help from their children). Therefore, when a child was showing signs of illness, mothers worried about not having enough cash, as one woman expressed during a focus group discussion:

*Your child may be sick, and you have nothing in your pocket in order to treat him. As your mother used to go and get plants to heal you, you will also go to find the same plants, boil them, and then wash your child. Meanwhile, you will continue to look for money, so that if the child does not get cured, you go to the clinic.*

Except for herbal medicines based on plants that they could themselves collect in the bush, all forms of treatment have a direct cost. However, with the traditional healers it was possible to negotiate the payment to be made as food products or to obtain credit. At the health facility this was not possible in spite of the fact that a proportion of the monthly income of the health facility is supposed to be set aside to cover for the poorest community members. Yet few villagers knew of the existence of this fund, the local health committee members did not push for its utilization and the health workers said that they were unsure about its inclusion criteria. The indirect costs included resources to cover for lost income and transportation. Vulnerability in this domain stemmed from the fact that few people had savings to pay for healthcare with. The “*meanwhile, you will continue to look for money”* statement covers a process of social interaction using culturally specific tactics to borrow money to cover for unknown costs. Time being spent on mobilizing resources meant delays in acting on a decision to seek help at health facilities, as seen in the case of Adrienne’s seemingly paradoxical decision to keep her cabaret open rather than accepting an offer of transportation on a day when her child was ill. She knew that failing to open her cabaret would cost her in terms of lost income and a missed opportunity for maintaining social relations. These concerns had to be weighed against each other in her decision-making process regarding when to go to the health facility.

### Coping strategies: seeking hopeful directions

Our data suggest that when mothers discover a symptom in the child, they are alert and take it seriously. They immediately try different modes of treatment, depending on what is convenient in the situation, as Fatoumata, a young mother of 25 years, said:

*Normally, if the child gets sick, I’ll start with traditional medicine, and I go to the pharmacy [at the dispensary] to buy paracetamol. If he is still sick after this, I'll go to the hospital* (healthcare center*) for help. If he is not cured after this, I now return to traditional medicine.*

Their responses can be divided into three forms of action: (i) home-treatment, (ii) consultation with a traditional specialist, and (iii) consultation with a biomedical specialist at the healthcare center. Home-treatment typically consisted of paracetamol, a few tablets of antimalarial medicines or plants that were boiled into a liquid that was used to wash the child with. What is noteworthy is how mothers seemed unsurprised by treatments not working and their swift move toward alternative solutions in successive sequences of folk/traditional and biomedical forms of treatments that were underpinned by colliding medical paradigms [31]. This flexible mode of action seems to reflect a pragmatic recognition of the uncertainties involved in care-seeking from both the traditional and the biomedical system:

*Here, when your child is sick you pray and ask God to give good health. You also find leaves, boil them, and wash the child with that. It may work, or it may not work. If it doesn’t, you will bring the child to the healthcare center. And if the child doesn’t get better, you will return to prayers.*

Mothers did little to know the exact diagnosis. What mattered was the possibility of action, rather than knowledge and reasoned recognition [32]. Mothers were solution-oriented in that they were concerned with bringing immediate relief to the child by keeping possibilities open rather than investing everything in one form of treatment that might turn out to be inefficient.

According to the conventional norm, a woman must ask her husband or the head of the household for permission before initiating any action outside of home [33]. Yet our data suggest that decisions about care-seeking for sick children were often made by women alone. This was in particular the case among women in town who often benefitted from a higher level of education and economic independence than women in villages. In our observations they had room to maneuver but they lacked the means to achieve long-term solutions. First, mothers often interrupted a biomedical treatment prematurely, either because the child’s condition improved leading them to save medicine for the next time, or because they ran out of money. As in Adrienne’s case, this entailed a risk of the child relapsing into new episodes of the same sickness. Interruptions in treatment exposed the mothers to criticism by the health professionals. In focus groups as well as in our observations, mothers were subject to criticism when they brought a child back to the dispensary with rapidly progressing fever. If the health workers suspected women of having interrupted the prescribed treatment they were treated as irresponsible mothers. In private conversations the health workers would blame the fathers for being unwilling to spend money on child health, yet they raised their voice against the mothers. In spite of their recognition of the widespread poverty in the village, the nurses expressed frustration about what they perceived as the villagers’ reluctance to invest time and money in treatment of children’s sicknesses.

### Routinization of the clinical encounter

Although state-organized healthcare in rural Burkina Faso prioritizes mother-child illness and has a particularly strong focus on malaria, as it is an endemic disease in the country, it is also characterized by poor infrastructure and lack of diagnostic equipment and supplies, which otherwise underpins up-to-date biomedical treatment. In the absence of advanced diagnostic equipment, healthcare is performed in standardized ways [29], following a set of routinized procedures. This includes arriving at a diagnosis making use of the clinical gaze and, in cases of fever, applying the rapid diagnostic test (if available). This is followed by standardized prescription practices based on the policy of treating malaria presumptively and finally instructing the patient (or the companion) in how to take the medicines at home. At the village dispensary, the nurse would place her hand on the forehead of the child. If the body was hot, a rapid malaria test would be performed and if it was burning with fever, the nurse might get up and take the piece of cloth that the child was wrapped in and soak it in water in an attempt to cool the child’s sickened body. Regardless of the test result, the mother would be given a prescription of anti-malarials and paracetamol, either as syrup if she was perceived as being able to afford it, or the cheaper tablets, which were difficult for a child to swallow. The administration of drugs would be explained verbally to the mothers who could rarely read the instructions on the prescription. We have noticed these standardized practices during the many hours of observations of clinical encounters at the rural health facilities. This *routinization* of the clinical encounter may contribute to the risk of facility based misdiagnosis, missing out on co-morbidity and delays in the treatment of other potentially life-threating diseases. These possible delays were, however, not verbalized by the health workers, who were more concerned with mothers’ responsibility for delays in reaching the facility.

At prenatal health-talks women were informed by the midwives about their responsibility for promptly taking a sick child to the healthcare center. However, once they were there, their chances of being seen by a nurse were uncertain, as can be seen in this quote from a focus group where a woman explains her frustration over unexplained waiting:

*You decide to go to the hospital but if you don’t leave very early in the morning you will not get a consultation. So you might have left your home at five in the morning and you might need to wait until one o’clock, and even then no one has paid attention to you. And you just stand there, waiting.*

Furthermore, the social organization of healthcare at the rural dispensaries was characterized by a strong hierarchy between the health workers and the patients. The health workers’ discretionary power was being used to organize who would be seen and when, in ways that were unclear to the women. In our observations from three different healthcare centers, waiting was not necessarily an outcome of a high influx of patients. It also occurred in situations where the health workers were absent because of leave, training or farming. Women knew that social relations were decisive for their chances of attracting the attention of the health workers. To be on good terms with the health workers women tried to “read” and mimic the social codes at the dispensary in a set of embodied tactics. They became a different version of themselves, timid, and refrained from asking for clarifications when they did not understand instructions about the administration of prescribed medicines. In our observations women navigate on the basis of personal, trusted contacts. Therefore, as soon as they entered the premises of the center they scanned the place for any familiar face among the staff. The health workers stood out as educated individuals who had mastered a biomedical language and practiced a set of social codes, which were different from local customs. For example, civility in the village included extensive greetings. By contrast, in our observations, health workers would walk rapidly past people waiting in front of the consultation room without greeting them. Enacting public healthcare in these ways contributed to the accumulation of vulnerability of the mothers.

According to our data, mothers were aware that they would be required to pay a fee but uncertain about how much, and about which services were free and when they were expected to offer a gift to the health worker in recognition of services delivered, and this confusion added to women’s mistrust in health workers. In the case of Adrienne, she suspected the nurses of having made her pay more than she should for medicines and she had decided to use the healthcare center 6 km away by bush road despite the fact that she had to borrow a bike to get there. Poor communication in the clinical encounter did not only concern child health services. For example, after the birth of her twins Adrienne had enquired about birth control out of fear of a new pregnancy but when the nurse informed her that there was a fee for injectables she simply left. One year later she was pregnant again.

## Discussion

### The dynamic effects of vulnerabilities

Drawing on qualitative methods, this article explored the relationship between women’s intentions to use modern health services and their social strategies and cultural practices to act on that intention. Through a detailed analysis of ethnographic data we have shown how mothers juggle multiple therapy paths. According to our findings women in this study wished to use public healthcare if they could mobilize the required resources. Yet, we also found that none of the women stayed within one health system (home, traditional and modern). They either combined treatment forms or shifted between systems, depending on their ability to afford biomedical medicines (not knowing the cost horizon) or interrupted treatments when they found progress to be achieved. Our study unfolds two patterns of healthcare seeking practices: a hierarchical resort in which women consult the local health facility first and continuously; and a simultaneous resort where they try out different options at the same time. These different resorts seemed to be associated with assessment of the type of sickness, course of the sickness as well as with available means to invest in treatment. In a quantitative study about utilization of public healthcare in rural Burkina Faso, Mugisha and colleagues [34] show that the factors that influence patient demand for healthcare (financial barriers) are not the same as those that enable retention (perceived quality of care, trust in the competency of the provider). The authors compare different independent factors influencing choice of health system and retention for successive illness episodes using variables such as household income, position in household and level of education. The results suggest that the likelihood of patients shifting from biomedical healthcare to traditional or home-treatment is connected to perceived poor quality of services. Our study corroborates these findings and in addition, show that the interconnectedness of the factors that hamper care-seeking, be it demand or retention, play a bigger role than so far recognized. We found that in the lived reality of mothers it was not the isolated obstacles to healthcare that conditioned the health seeking process but rather the continuous sequences of interconnected illness episodes. In the case of Burkina Faso, where malaria is endemic, the effect of cumulative processes of repeated malaria episodes seems particularly fit to focus on the accumulation of difficulties that people encounter when they reach out for public healthcare.

We argue that in a context of social inequity and poverty women’s actions or inactions are conditioned by their accumulated vulnerabilities. For most mothers in our study, child sickness came as a sequence of improvement, followed by relapses of fever, anemia and malnutrition, which the case of Adrienne’s daughter illustrates. Mothers’ experiences of treatment “not working” is captured in Kleinman’s concept of “treatment sans healing” [35]. Caring for a sick child had become a routine based on uncertainty. The hardship they went through when their juggling failed led to emotional and physical exhaustion. In order to maintain hope, mothers acted in what can be conceptualized as a “subjunctive mode”: a mode of action where healthcare-seeking is being practiced with a maximum of possibilities being kept open [36]. The subjunctive mode activates hopes of positive outcomes as it underpins possibility rather than certainty and probability. Women in this study rarely experienced child health problems being fully solved. Thus, there was neither time nor resources to be spent on prevention, so mothers invested in immediate relief rather than long-term solutions.

Although standards are often endorsed by experts, they may, as emphasized by Timmerman and Epstein, “come to function as an alternative to expert authority – a way of embedding authority in rules and systems rather than in credentialed professionals” [29]. The routinization of the clinical encounter with its standardized procedures of making a diagnosis and prescribing medicines may actually challenge the aspirations of reducing the high rates of morbidity and mortality among children in rural Burkina Faso. The primary level of the healthcare system is neither equipped nor primed to take the complexity of co-morbidities and accumulated vulnerabilities into account. Data from our study show that the mothers are pragmatically aware of the limitations of the services offered at the rural dispensaries – in the sense that they shift quickly from home treatment to public healthcare treatment to traditional treatments, knowing very well that “children get sick all the time”.

We would like to mention a limitation of this study. Due to its anthropological approach we have foregrounded one empirical case in order to analyze in detail the nature of accumulated vulnerabilities that women in this context encounter. Thereby we run the risk of giving overly weight to experiences of one informant. To address this our data analysis has been rigorous and our findings concerning health-seeking patterns are based on a combination of this case validated against the observations yielded among the group of women that we followed intensely over a prolonged period. Our findings apply to this specific study area, however, they can be considered relevant for similar context in the sub-region and for studies that wish to study quality of care in a context of material poverty.

## Conclusion

In Burkinabe public healthcare, children (and their mothers) come first. At least that is how the state-sponsored health service is designed [37, 38]. Child health services are exempted from out-of-pocket payments and the most important health worker performance targets are connected to provision of maternal and child services. Yet if we take a closer look at the way mothers struggle, and fail, to receive care we see that service delivery could be organized in a manner that responds better to their needs. Using the concept of accumulated vulnerabilities as proposed by Ribera and Hausmann-Muela [28] we found that women prefer modern healthcare in case of children’s sicknesses but different and interacting forms of vulnerabilities hinder them in achieving just that. Mothers actively try to create the best possible situation for their children’s recovery but the sporadic character of their actions weakens the chances of treatment success. We have shown that these mothers can be understood as intentional subjects, whose practices were apprehensible as a way of acting that kept possibilities open, maintaining hope and steering through situations of vulnerability. In addition, the poor equipment and routinization of the clinical encounter at the rural dispensaries fails to address the complexity of child illnesses in rural communities where malnutrition and various infectious diseases create a dangerous cocktail.

The immediate implications of our findings for healthcare policy are that interventions to strengthen healthcare utilization, with the overall aim of reducing child morbidity and mortality, should focus on improving the conditions that enable both access to treatment and retention. First, removal of user fees would increase utilization of child healthcare and child survival by the poorest [39–43]. Secondly, investment in better quality of care through improved social relations between healthcare providers and users could establish a trust base enabling higher retention. Careful attention to the negative aspects of routinized health care practices should also be addressed. This would require investment in health worker capacity to mobilize interpersonal, cultural and technical competences to overcome difficulties in the rural facilities [44–49]. In the current situation, it is unlikely that merely investing in the upgrading of physical infrastructure would have an optimal effect on healthcare service utilization as long as the social relations between providers and patients are as complicated as they appear to be now.

### Ethics

Ethical permission for this study was granted by the Ethical Committee of the Ministry of Health and by the Burkina Faso Ministry of Scientific Research and Innovation and the Regional Directorate of Health [Reference number 2013-00000181/MRSI/SG/CNRST/DG/DS). Furthermore, with our interlocutors we explained the study purposes as well as the fact that it was possible and acceptable to withdraw from the interview, before obtaining their verbal consent.

### Consent to publish

Not applicable.

### Availability of data and material

Data will not be shared as it is stored in physical, including hand written notes, not digital form.

## Abbreviation

GRIL: Groupe de Recherche sur les Initiatives Locales
IRSS: Institut de Recherche en Sciences de la Santé
